# Wavelet Components of Photoplethysmography During Reactive Hyperemia: Absolute vs. Relative Metrics

**DOI:** 10.3390/biology14121727

**Published:** 2025-12-02

**Authors:** Henrique Silva, Nicole Lavrador

**Affiliations:** 1 Research Institute for Medicines (iMed.ULisboa), Faculty of Pharmacy, Universidade de Lisboa, Av. Prof. Gama Pinto, 1649-003 Lisbon, Portugal; 2 Department of Pharmacy, Pharmacology and Health Technologies, Faculty of Pharmacy, Universidade de Lisboa, Av. Prof. Gama Pinto, 1649-003 Lisbon, Portugal; nicole.lav45@gmail.com; 3 Biophysics and Biomedical Engineering Institute (IBEB), Faculty of Sciences, Universidade de Lisboa, Campo Grande, 1749-016 Lisbon, Portugal

**Keywords:** photoplethysmography, wavelet transform, spectral analysis, reactive hyperemia, skin microcirculation, endothelial function, vasomotion

## Abstract

Photoplethysmography (PPG) is a non-invasive optical technique that quantifies blood volume pulsations and enables assessment of skin microvascular dynamics during vascular challenges. Its complex waveform can be decomposed by wavelet transform (WT) into physiological frequency bands reflecting cardiac, respiratory, myogenic, neurogenic, endothelial NO-dependent and endothelial NO-independent activity. Spectral activity may be expressed as either absolute power or relative contribution, which can capture different aspects of microvascular regulation. This study compared both metrics of PPG signals during post-occlusive reactive hyperemia (PORH). PPG was recorded bilaterally in twelve healthy adults (21.6 ± 1.9 years) during a 10-min baseline, 5-min occlusion, and 10-min recovery. Spectral power and percent contribution were calculated for each band and phase using nonparametric statistics (*p* < 0.05). Occlusion markedly suppressed higher-frequency oscillations (cardiac, respiratory, myogenic, neurogenic) while enhancing endothelial activity. Hyperemia produced a rebound of respiratory and endothelial oscillations above baseline. In the contralateral limb, occlusion induced a milder perfusion decrease, consistent with sympathetic vasoconstriction. Correlations between absolute and relative metrics were strong for cardiac, respiratory, and myogenic components (ρ > 0.80, *p* < 0.01), but weak or absent for endothelial bands. Absolute power primarily reflected perfusion magnitude, whereas relative contribution represented spectral redistribution among regulatory mechanisms. These metrics are complementary yet non-equivalent, underscoring the need for methodological consistency in the physiological interpretation of wavelet-derived PPG analyses.

## Simple Summary

This study investigated how skin microvasculature responds to a brief interruption of blood flow, a physiological test known as post-occlusive reactive hyperemia. Using photoplethysmography, a non-invasive optical technique that tracks pulsations in skin blood volume, we analyzed the rhythmic oscillations that reflect different mechanisms regulating microvascular tone. A wavelet-based approach allowed us to decompose the signal into frequency bands associated with cardiac, respiratory, myogenic, neurogenic, and endothelial activity, and to compare two ways of expressing their magnitude: absolute power and relative contribution. During occlusion, higher-frequency components related to cardiac and neural control were strongly suppressed, while the slower endothelial components became more prominent. Upon reperfusion, oscillations in the respiratory and endothelial ranges rebounded above baseline levels. The comparison between absolute and relative measures showed that these metrics provide complementary but non-equivalent information: absolute power primarily reflects the amount of blood flow, whereas relative contribution reveals changes in the distribution of regulatory influences. These findings highlight the importance of methodological consistency and support the use of wavelet analysis for improving physiological interpretation and non-invasive assessment of microvascular function in research and potential clinical applications.

## 1. Introduction

Photoplethysmography (PPG) is a non-invasive optical technique widely used in the clinical setting, most notably as the basis of pulse oximetry to estimate arterial oxygen saturation, a key index of cardiorespiratory function [1]. For experimental purposes, however, it has been used to assess skin microcirculation [2,3], to quantify aortic pulse-wave velocity [4]. Recent advances in miniaturization have enabled PPG sensors to be incorporated into wearable devices, broadening their applications for the continuous monitoring of physiological parameters and health-related metrics [5]. The PPG waveform contains information of multiple physiological origins and time scales, which has motivated the application of nonlinear and multiscale analytical tools to extract novel physiological descriptors of vascular regulation.

Spectral analysis has been frequently applied to PPG, most often using the fast Fourier transform (FFT). The continuous wavelet transform (CWT) provides better time–frequency localization, allowing decomposition into six spectral bands [6]. These components refer to the cardiac [2–0.6 Hz], respiratory [0.6–0.145 Hz], myogenic [0.145–0.052 Hz], neurogenic [0.052–0.021 Hz], endothelial nitric oxide (NO)-dependent [0.021–0.0095 Hz] and endothelial NO-independent [0.0095–0.0050 Hz] bands, with each displaying a specific frequency range of activity. Fourier-based decomposition is also the basis of pulse rate variability, an analogue of heart rate variability used to assess autonomic modulation [7]. Despite its increasing use, several issues limit the reproducibility of wavelet analysis in biological signals [8]. These include the need to select time–frequency regions with adequate statistical support [9], the limited time–frequency resolution that blurs component boundaries, the possibility of cross-component interactions, the impact of preprocessing choices, and the diversity of reporting metrics. Previous studies have expressed results either in absolute terms (power or amplitude) or in relative terms (percent contribution or normalized spectral area) [10,11,12,13,14], which carry very different interpretations. Unfortunately, few studies compare absolute and relative metrics of perfusion signals, limiting our ability to interpret these indices in well-defined physiological contexts such as reactive hyperemia. Determining whether absolute and relative wavelet-based metrics capture overlapping or distinct physiological processes is essential to harmonize analytical approaches and ensure consistent interpretation of PPG-derived microvascular indices.

The present study aims to compare absolute and relative wavelet-derived metrics of raw PPG signals during a standard post-occlusive reactive hyperemia (PORH) protocol, contrasting the occluded and contralateral limbs to explore their utility in the assessment of skin perfusion dynamics.

## 2. Materials and Methods

### 2.1. Subjects

Photoplethysmography signals were analyzed from twelve healthy volunteers (21.6 ± 1.9 years old). All subjects provided written informed consent. The inclusion criteria were male or female (non-pregnant), between 18 and 35 years old, non-obese (body mass index < 30 kg/m^2^), and with blood pressure within the non-hypertensive range defined by the European Society of Cardiology/European Society of Hypertension (ESC/ESH) 2018 classification [15]. The defined exclusion criteria were a current or past history of cardiovascular, metabolic, neurologic or psychiatric diseases, and taking vasoactive medications (including contraceptives) or dietary supplements. Subjects were asked to refrain from drinking caffeinated beverages and from performing physical exercise 12 h prior to the procedures. Subjects were instructed to fast for 2–4 h before performing the procedures to minimize any potential effects on sympathetic drive and endothelial activity, including the vasodilatory effect of insulin [10,11]. Table 1 summarizes the characteristics of these subjects. The study was approved by the local ethics committee and followed the recommendations of the Declaration of Helsinki and subsequent amendments for studies conducted in human subjects [16].

### 2.2. Procedure

Experiments were conducted in a temperature- and humidity-controlled room (22–24 °C; 40–60%). The PORH protocol consisted of three phases: 10 min baseline, 5 min arterial occlusion at 200 mmHg, and 10 min recovery. Cuff inflation and deflation were completed within 10 s.

### 2.3. Technologies

Photoplethysmography is an optical technique that detects changes in tissue light absorption caused by pulsatile variations in microvascular blood volume. A light source illuminates the skin, and the backscattered light returning to the photodetector fluctuates with each cardiac cycle as local blood volume changes: during systole, arterial distension increases local absorption, whereas during diastole, smaller vessel diameter allows greater reflection [3,5]. The resulting signal comprises a slow-varying direct-current (DC) component, reflecting venous blood volume and tissue optical properties, and a pulsatile alternating-current (AC) component, corresponding to rapid arterial inflow changes with each heartbeat. The AC component is sensitive to dynamic perfusion and sympathetic vasomotor tone, while the DC component represents slower adjustments in venous filling and microvascular compliance. Because the PPG waveform contains oscillations arising from multiple physiological processes at distinct time scales, wavelet decomposition enables these components to be separated into frequency bands representing cardiac, respiratory, myogenic, neurogenic, and endothelial activity. This makes PPG particularly suitable for characterizing microvascular regulation during vascular challenges, such as PORH.

In this study, PPG signals were recorded in reflectance mode from the palmar surface of the index finger of each hand, using identical sensors placed symmetrically to ensure comparable measurement depth and microvascular density across limbs. The sensors were connected to a BITalino microprocessor board (PLUX Biosignals, Lisbon, Portugal). Each sensor used a green LED (530 nm) with 2.3 mm emitter–detector separation, coupled to 10-bit acquisition channels.

Raw signals were acquired at 100 Hz using the OpenSignals (r)evolution software (PLUX Biosignals, Portugal) and subsequently analyzed in Matlab R2015a (MathWorks, Natick, MA, USA). Skin blood flow was estimated as the value at the systolic peak of a given pulse wave minus the value at the onset point of the following pulse wave, and expressed in arbitrary units (AU).

### 2.4. The Wavelet Transform

We used the continuous wavelet transform (CWT) to obtain time-resolved spectra of PPG signals [17], based on a Matlab toolbox (http://noc.ac.uk/using-science/crosswavelet-wavelet-coherence, accessed on 19 November 2025). For a signal *x*(*t*) sampled at Δ*t*, the CWT is:

$$WT\left(s,\tau \right)=\frac{1}{\surd s}\int x\left(t\right)\psi^{*}(\frac{t-\tau }{s})dt$$

with the complex Morlet mother wavelet

$$\psi (t)=\pi^{-\frac{1}{4}}exp\left(iw_{0}t\right)exp\left(-\frac{t^{2}}{2}\right),$$

using $\omega_{0}=6$, a standard choice that provides good joint time–frequency localization for cardiovascular oscillations. Scales were defined as:

$$s_{j}=s_{0}2^{j\delta_{j}}\;\mathrm{for}\;j=0,\;\ldots ,\;j_{1}$$

where *j* is the scale index, *δ*_*j*_ is the scale resolution (here 1/12), and *s*_0_ = 2Δ*t* is the minimum scale. Periods were obtained from scales via the Morlet Fourier factor:

$$Period=s\times \frac{4.\pi }{\omega_{0}+\sqrt{2+\omega_{0}^{2}}}\approx 1.033\;s$$

and frequency was defined as f = 1/period. Signals were zero-padded and the cone of influence (COI) was computed. All time points for which the local period exceeded the COI boundary were excluded from subsequent averaging. The scalogram was defined as:

$$P\left(s,\tau \right)={\left|W(s,\tau )\right|}^{2}.$$

Wavelet power was characterized using two metrics—(a) absolute power: the activity of each physiological component was quantified as the area under the time-averaged wavelet power spectrum (AUC of $|W(s,\tau ){|}^{2}$) within its predefined frequency band. This yields an integrated measure of spectral energy (AU^2^); (b) relative contribution: the percent contribution of each component was computed as:

$$\mathrm{Contribution}=\frac{P_{\mathrm{band}}}{\sum_{\mathrm{bands}}P_{\mathrm{band}}}\times 100$$

### 2.5. Statistical Analysis

Raw signals were analyzed without filtering to preserve physiological content. No data segments were removed, as wavelet decomposition is sensitive to discontinuities. Instead, stable time epochs were selected away from transitions or minor artifacts, ensuring minimal artifact influence within the COI. Three stable signal epochs were selected for analysis: 6–9 min (baseline), 11–14 min (occlusion), and 16–19 min (hyperemia). The activity of each spectral component was quantified in terms of absolute power and relative contribution. Spectral power for each component was quantified as the area under the time-averaged squared magnitude of the wavelet coefficients within its frequency range. This provided an integrated measure of spectral energy. The percent contribution of each component was obtained by dividing its power by the total power across all components and multiplying by 100%. This expresses the proportion of total spectral energy for each physiological oscillator. This preserves the energetic interpretation of power spectral density and enables direct comparison with Fourier spectra. Normality was assessed using the Shapiro–Wilk test, and as variables were not normally distributed, non-parametric tests were applied. Phase and limb comparisons were performed with the Wilcoxon signed-rank test for paired samples. Correlations were evaluated using Spearman’s rank test. A two-tailed *p*-value < 0.05 was considered statistically significant.

## 3. Results

**Figure 1:**
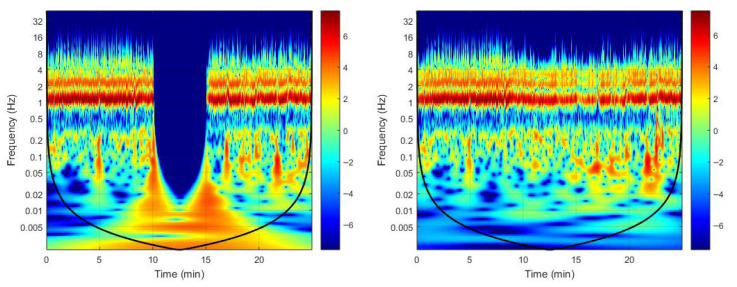
Time–frequency representation of the photoplethysmography (PPG) signal obtained by continuous wavelet transform in the occluded (**left** panel) and contralateral (**right** panel) arms of a representative subject (male, 22 years). Power values (color scale, log-normalized) reflect the relative contribution of oscillatory components over time. The black curve marks the cone of influence, outside which edge effects become significant. Downward frequency cascades can be observed at approximately 10 min (cuff inflation) and 15 min (cuff deflation).

**Figure 2:**
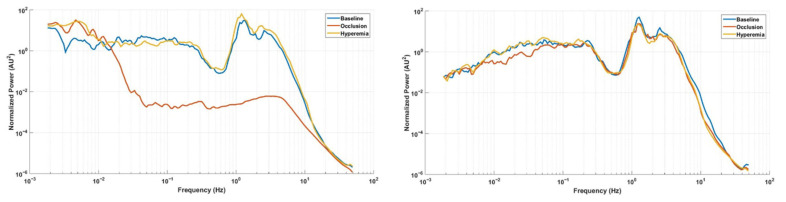
Median (N = 12) wavelet frequency spectra of PPG signals recorded from the test and contralateral limbs. The wavelet power at each frequency was normalized by the total signal variance. Log–log scaling highlights the redistribution of power across physiological frequency bands during the vascular response to ischemia and reperfusion.

Table 2 and Table 3 present the median (IQR) values of skin blood flow (AU), spectral power and percent contribution for all frequency components in the test (occluded) and contralateral limbs. Figure 1 shows the redistribution of time–frequency energy across the three phases, and Figure 2 provides the corresponding median wavelet spectra for both limbs, highlighting the suppression of high-frequency components during occlusion and the rebound of slow endothelial oscillations during hyperemia.

**Spectral power.** During occlusion, spectral power in the test limb decreased significantly in the cardiac (*p* = 0.003), respiratory (*p* = 0.005), and myogenic (*p* = 0.002) bands. In contrast, NOi power increased (*p* = 0.002), whereas NOd showed a non-significant rise. During hyperemia, spectral power exceeded baseline values for the respiratory (*p* = 0.041), NOd (*p* = 0.019), and NOi (*p* = 0.010) components.

In the contralateral limb, occlusion produced a significant reduction in skin blood flow and in sympathetic (neurogenic) power (*p* = 0.049). The relative change from baseline to occlusion was greater in the test limb for the cardiac (*p* = 0.002), respiratory (*p* = 0.023), myogenic (*p* = 0.005), and neurogenic (*p* = 0.034) bands. In contrast, it was smaller for the NOi band (*p* = 0.002).

**Percent contribution.** Occlusion caused a significant decrease in the relative contribution of the cardiac (*p* = 0.002), respiratory (*p* = 0.002), myogenic (*p* = 0.002), and neurogenic (*p* = 0.014) components. In contrast, NOd (*p* = 0.010) and NOi (*p* = 0.002) contributions increased. No significant changes were observed in the contralateral limb. The NOi band consistently showed higher relative contribution in the test limb compared with the contralateral (*p* = 0.002 for all phases). During occlusion, the contralateral limb showed higher contribution in the cardiac (*p* = 0.002), respiratory (*p* = 0.002), myogenic (*p* = 0.002), and neurogenic (*p* = 0.010) bands. Conversely, NOd (*p* = 0.004) and NOi (*p* = 0.002) contributions were lower. From baseline to occlusion, changes were again greater in the test limb for the cardiac, respiratory, myogenic, and neurogenic bands, but smaller for NOd and NOi. During hyperemia, the contribution of the respiratory (*p* = 0.031), myogenic (*p* = 0.002), neurogenic (*p* = 0.019), and NOi (*p* = 0.002) bands increased significantly in the test limb.

Overall, the results reveal suppression of high-frequency activity during occlusion and rebound of slow endothelial oscillations during hyperemia, most evident in the occluded limb. To assess whether both metrics captured the same phenomena, we computed the correlations between absolute power and relative contribution changes for the baseline-to-occlusion transition (Table 4).

## 4. Discussion

In this study, we highlight the differences between absolute and relative metrics used to assess the activity of the various components of raw PPG signals. These components are observed in the same spectral regions previously described for laser Doppler flowmetry (LDF) [18]. Current results suggest that they are able to record the same phenomena, although in different expressions, due to the distinct biophysical profiles of those techniques [19,20,21,22]. Currently, PPG is being considered as a possible replacement for LDF, especially for measuring low-frequency phenomena [23]. Assuming that PPG can detect the same physiological oscillators as LDF, we considered that their spectral positions remain unchanged. However, the physiological origin of these components was originally inferred from changes observed in LDF spectra of human subjects following specific experimental manipulations or pharmacological interventions. The 2–0.6 Hz spectral region, also termed the cardiac component, is attributed to the transmission of pulse-borne oscillations from the heart to the microcirculation [24], and aligned with the heart rate. Since the PPG waveform is largely dominated by cardiac pulsations, it is unsurprising that this component exhibited the highest spectral activity. The 0.6–0.15 Hz region coincides with the respiratory rate; therefore, it was termed the respiratory component [21,25]. The respiratory component is attributed to the transmission of ventilation-related oscillations to the microcirculation. Unlike ECG recordings, which exhibit baseline shifts caused by respiratory artefacts, PPG recordings display oscillations presumably linked to respiratory-induced variations in cardiac output, likely mediated by changes in venous return. The 0.15–0.052 Hz spectral region is attributed to the vasomotion activity of vascular smooth muscle, which was discovered when changes in blood pressure altered changes in microvascular caliber in vivo [4,26]. The neurogenic component is attributed to oscillations in vascular tone resulting from variations in sympathetic nerve activity and occupies the 0.052–0.021 Hz range. This spectral band decreases markedly in heated skin [27] as well as in denervated skin flaps [28], supporting its neurogenic origin. Finally, even lower frequencies were initially described as metabolic, having been assumed to be related to tissue metabolism [29]. The amplitude of the 0.0095–0.021 Hz region increases after cutaneous iontophoresis of acetylcholine. It is inhibited by N^G^-monomethyl-L-arginine and restored by arginine infusion, indicating an endothelial NO-dependent (NOd) origin [14,18]. The 0.005–0.0095 Hz region is unaffected by acetylcholine or aspirin (a cyclooxygenase inhibitor) and is therefore attributed to endothelium-derived hyperpolarization factors (EDHFs) [18]. An interesting yet puzzling aspect of wavelet analysis is the fact that the physiological systems responsible for generating these spectral bands can influence others. For example, the rate and depth of breathing are thought to influence the sympat-hetic control of vascular tone [30]. Also, anesthetic blockage of the brachial plexus attenuates both the neurogenic and NOd spectral regions [31], with the endothelial response likely reflecting the upstream reduction in neurogenic activity.

In this study, spectral activity was assessed using the area under the time-averaged wavelet power spectrum rather than amplitude-based metrics. This choice provides an integrated estimate of the total oscillatory energy within each physiological frequency band, reflecting both the strength and persistence of activity during each phase. Compared with amplitude or AUC-of-amplitude approaches, power integration offers greater robustness to transient fluctuations and noise, while preserving the physical relationship between signal variance and energy. Expressing spectral activity in energy terms ensures methodological consistency with conventional Fourier power spectral analysis, facilitating comparison across studies and enhancing the physiological interpretability of the results [13].

Our PPG spectra revealed marked differences between limbs, with spectral power closely reflecting the magnitude of skin blood flow. Accordingly, the PPG spectrum of the occluded limb exhibited a substantially lower amplitude compared with the contralateral limb. Across all phases, the cardiac component displayed the highest activity in both absolute power and relative contribution. In contrast, low-frequency components, particularly the endothelial bands, showed the lowest activity overall. This spectral organization contrasts sharply with that typically observed in LDF, where low-frequency components dominate the spectrum [20,21]. Overall, no baseline differences were detected in skin blood flow or spectral activities between limbs, with the notable exception of the endothelial NO-independent component. Although baseline conditions were assumed equivalent between sides, time–frequency maps revealed an apparent increase in NOi power on the test limb. This change likely resulted from a transient downward frequency cascade preceding occlusion (Figure 1). This pre-occlusion spreading of wavelet energy from higher to lower frequencies likely reflects non-stationary behavior of the signal rather than a genuine physiological asymmetry. In addition, the less than optimal signal duration might have been a contributing factor, since 30 min or more has been proposed as optimal to inspect low-frequency phenomena [13].

Post-occlusive reactive hyperemia is a complex vascular response involving multiple physiological effectors. During occlusion, capillary oxygen levels gradually fall due to continued metabolism and lack of arterial inflow. This hypoxic state promotes the endothelial release of nitric oxide (NO) [32], as well as the accumulation of tissue metabolites (carbon dioxide, lactate, hydrogen ions, adenosine) leading to dilation of vessels distal to the occlusion site [33,34]. The perception of discomfort or pain further enhances vasodilation through the release of vasoactive substances from sensory afferent fibers [35,36]. Myogenic-mediated dilation is also potentiated during occlusion due to perfusion restriction [37]. During the initial part of the reactive hyperemia spontaneous myogenic activity (i.e., vasomotion) disappears, until it resumes its original frequency [25]. Together, these mechanisms create favorable conditions for a sharp hyperemic surge once blood flow is restored, rapidly filling the previously collapsed vascular bed. As expected, arterial occlusion caused a pronounced reduction in skin perfusion. The PPG signal did not fall to zero, likely due to residual motion of trapped red blood cells and other minor artifacts [38]. Consequently, both spectral power and relative contribution decreased markedly for the cardiac, respiratory, myogenic, and neurogenic components. Interestingly, the power and contribution of the endothelial components increased significantly during occlusion. At first glance, this apparent rise could be interpreted as reflecting ischemia-induced activation of compensatory mechanisms, such as enhanced endothelial NO release [39]. However, because blood flow oscillations were largely suppressed, the apparent increase in endothelial activity is likely artefactual. Slow baseline drifts tend to dominate the spectrum when higher-frequency oscillations are dampened.

Although the 10-min baseline and recovery windows provided adequate resolution for the main low-frequency bands, as previously established in several microvascular studies using wavelet analysis [10,13,14,18], longer recordings could further improve the stability of very low-frequency (endothelial) components. However, excessively prolonged measurements in acral sites such as the fingertips may induce thermoregulatory vasoconstriction [40], which can obscure genuine flowmotion patterns. Moreover, unlike studies that apply the wavelet transform separately to shorter time segments [9,12], the present analysis computed a single continuous transform over the entire signal. This approach minimizes edge effects, as only the initial and final margins fall outside the cone of influence, thereby maximizing the effective signal length and allowing reliable estimation of low-frequency components within shorter total recording durations. The chosen window length therefore represents an optimized balance between physiological stability and analytical requirements for spectral resolution.

In the contralateral limb, skin blood flow also decreased significantly, although with a smaller magnitude than in the occluded limb. This “consensual” reduction in perfusion likely represents an anticipatory response aimed at maintaining arterial pressure. Occlusion simultaneously interrupts both arterial inflow and venous outflow, thereby reducing venous return and transiently lowering cardiac preload. The contralateral vasoconstriction observed here may thus serve to counteract this fall in blood pressure. In this limb, the reduction in perfusion was accompanied by a decrease in the absolute power of all spectral components. Interestingly, the relative contributions did not follow the same trend, showing a selective reduction in the respiratory and NO-dependent endothelial bands. Mechanistically, this contralateral decrease in blood flow is likely mediated by an increase in sympathetic activity, as suggested by the concurrent rise in myogenic and neurogenic components. The unexpected increase in respiratory activity, absent in the occluded limb due to PPG suppression, may reflect enhanced ventilation during occlusion. This would be consistent with activation of brainstem respiratory centers by discomfort or pain [41]. Such contralateral vasoconstriction should be accounted for when interpreting unilateral PORH tests.

We tested the correlations between absolute and relative metrics (power and percent contribution) to determine whether they capture the same physiological phenomena or instead reflect independent aspects of vascular activity. In the test limb, the cardiac, respiratory, myogenic, and neurogenic components displayed strong correlations, indicating that both metrics primarily reflect actual perfusion. In contrast, endothelial components showed weak or absent correlations. This suggests that relative contribution behaves differently, possibly reflecting regulatory mechanisms or low-frequency artifacts. In the contralateral limb, endothelial correlations were stronger, likely due to lower noise and a smaller reduction in perfusion. No significant correlation was found for the respiratory band, whose power decreased while its relative contribution increased. This apparent paradox may result from a redistribution of spectral energy. Part of the power from the adjacent myogenic band may have been reassigned to the respiratory range. Similarly, neurogenic power increased while its contribution decreased, which may also reflect partial spectral overlap with myogenic activity. Although waveform morphology can vary across anatomical sites, both sensors were positioned on homologous finger regions, ensuring comparable microvascular density and optical sampling conditions. Therefore, site-dependent influences on the spectral results are expected to be minimal.

Taken together, these findings indicate that absolute and relative wavelet-derived metrics convey fundamentally different information, limiting direct comparison across studies. Absolute power mainly reflects perfusion magnitude, whereas relative metrics capture the redistribution of regulatory influences across frequency bands. Both are valuable but fundamentally distinct descriptors of vascular dynamics. This distinction underscores the importance of methodological consistency and cautious physiological interpretation in future applications of wavelet analysis.

From a translational standpoint, understanding how absolute and relative wavelet-derived metrics behave under different physiological conditions may improve the clinical and technological applicability of PPG. In pathological contexts such as aging, hypertension, or diabetes, endothelial and neurogenic oscillations are often blunted [42,43,44,45], potentially altering the balance between spectral components. Identifying whether these changes are better captured by absolute or relative metrics could therefore enhance the sensitivity of PPG-based assessments of microvascular dysfunction. Moreover, as wearable devices increasingly rely on PPG sensors, standardized analytical frameworks are needed. These could facilitate their use in long-term monitoring of microvascular health.

From a practical perspective, these findings may contribute to improving wearable health monitoring and early detection of vascular dysfunction. By distinguishing between absolute and relative wavelet-derived metrics, it becomes possible to separate changes in overall perfusion from shifts in regulatory dynamics, an essential step for reliable interpretation of PPG signals in real-world settings. Incorporating such analytical approaches into wearable devices could enhance their capacity to detect microvascular alterations associated with aging, hypertension, or metabolic disorders, thereby supporting preventive and personalized medicine.

## 5. Conclusions

Wavelet analysis of PPG signals during PORH reveals that absolute and relative metrics capture distinct aspects of microvascular behavior. Absolute power primarily indexes perfusion, whereas relative contribution reflects the redistribution of regulatory influences across frequency bands. This underscores the need for methodological consistency and cautious physiological interpretation. Future studies should clarify whether relative metrics provide added sensitivity to specific regulatory perturbations, such as endothelial dysfunction or autonomic imbalance.

## 6. Limitations

This proof-of-concept study has several limitations. It was conducted in a small, homogeneous cohort of young adults to minimize interindividual variability. While this approach strengthens internal consistency, it limits extrapolation to broader age ranges and clinical populations. The findings therefore represent physiological trends under tightly controlled experimental conditions rather than population-level variability. Wavelet-based indices were assessed only in healthy skin, without evaluation of reproducibility across recording sites or sessions. Although procedural and environmental conditions were carefully standardized, minor temperature fluctuations or motion artifacts cannot be entirely excluded. The study was not powered for inferential comparisons between sexes or clinical subgroups, as its primary goal was to characterize the relationship between absolute and relative wavelet-derived metrics within a controlled physiological framework. Finally, although the 10-min baseline and recovery windows provided adequate resolution for the main low-frequency bands, longer recordings might improve the stability of very-low-frequency (endothelial) components, albeit at the cost of increased thermoregulatory vasoconstriction in acral skin. Future studies including larger and more diverse samples are warranted to confirm the generalizability of these observations.

## Figures and Tables

**Table 1:** Characteristics of subjects (means ± standard deviations).

	Total	Males	Females
N	12	6	6
Age (years)	21.6 ± 1.9	21.2 ± 1.2	22.0 ± 2.4
Body mass index (kg/m^2^)	23.3 ± 4.2	21.8 ± 2.8	24.6 ± 5.0
Systolic blood pressure (mmHg)	115.2 ± 8.4	118.0 ± 4.5	112.8 ± 10.5
Diastolic blood pressure (mmHg)	76.2 ± 8.9	73.0 ± 8.6	78.2 ± 9.5
Fasting period (h)	3.4 ± 0.8	3.4 ± 0.7	3.3 ± 0.1
Menstrual cycle duration (days)	-	-	29 ± 1
Menstrual cycle day	-	-	5 ± 2

**Table 2:** Median (IQR) skin blood flow (AU) and wavelet spectral power (AU^2^) of the physiological components obtained from the raw PPG signals in the test and contralateral limbs. (* *p* < 0.05).

Component		Test (T)	Contralateral (C)	*p*-Value (T vs. C)
Skin blood flow (AU)	Baseline	443.4 (37.3; 781.1)	461.5 (49.5; 759.7)	0.155
	Occlusion	12.9 (6.3; 63.3)	456.9 (62.9; 724.8)	0.003 *
	Hyperemia	508.9 (172.9; 735.8)	495.8 (59.4; 672.9)	0.041 *
	ΔII-I (%)	−96.4 (−99.2; −5.2)	−8.0 (−48.6; 59.6)	0.002 *
	*p*-value (II vs. I)	0.003 *	0.006 *	-
	*p*-value (III vs. I)	0.084	0.366	-
Cardiac	Baseline	1.1 × 10^11^ (2.3 × 10^9^; 9.1 × 10^11^)	1.6 × 10^11^ (2.9 × 10^9^; 6.1 × 10^11^)	0.308
	Occlusion	2.1 × 10^7^ (3.5 × 10^6^; 2.3 × 10^9)^	1.2 × 10^11^ (3.6 × 10^9^; 4.9 × 10^11^)	0.002 *
	Hyperemia	1.4 × 10^11^ (2.7 × 10^10^; 7.6 × 10^11^)	1.5 × 10^11^ (4.8 × 10^9^; 3.6 × 10^11^)	0.041 *
	ΔII-I (%)	−100% (−100; 55)	−46% (−82; 510)	0.002 *
	*p*-value (II vs. I)	0.003 *	0.071	-
	*p*-value (III vs. I)	0.638	0.049 *	-
Respiratory	Baseline	1.1 × 10^10^ (8.5 × 10^8^; 4.6 × 10^10^)	1.5 × 10^10^ (2.0 × 10^9^; 2.3 × 10^10^)	0.433
	Occlusion	1.0 × 10^7^ (3.7 × 10^6^; 3.5 × 10^9^)	1.1 × 10^10^ (1.2 × 10^9^; 4.6 × 10^10^)	0.002 *
	Hyperemia	1.9 × 10^10^ (5.2 × 10^9^; 4.1 × 10^10^)	1.6 × 10^10^ (2.9 × 10^9^; 7.4 × 10^10^)	0.875
	ΔII-I (%)	−100% (−100; 57)	−26% (−51; 130)	0.023 *
	*p*-value (II vs. I)	0.005 *	0.638	-
	*p*-value (III vs. I)	0.041 *	0.433	-
Myogenic	Baseline	3.0 × 10^10^ (3.3 × 10^9^; 7.6 × 10^10^)	2.7 × 10^10^ (3.6 × 10^9^; 9.1 × 10^10^)	0.530
	Occlusion	8.0 × 10^6^ (2.4 × 10^6^; 2.6 × 10^9^)	2.3 × 10^10^ (7.0 × 10^9^; 5.1 × 10^10^)	0.002 *
	Hyperemia	2.5 × 10^10^ (8.1 × 10^9^; 1.3 × 10^11^)	3.1 × 10^10^ (5.2 × 10^9;^ 1.2 × 10^11^)	0.937
	ΔII-I (%)	−100% (−100; −41)	−25% (−72; 392)	0.005 *
	*p*-value (II vs. I)	0.002 *	0.099	-
	*p*-value (III vs. I)	0.060	0.638	-
Neurogenic	Baseline	2.6 × 10^10^ (1.8 × 10^9^; 7.2 × 10^10^)	3.0 × 10^10^ (2.0 × 10^9^; 5.6 × 10^10^)	0.638
	Occlusion	6.7 × 10^8^ (1.4 × 10^8^; 1.2 × 10^10^)	7.3 × 10^9^ (2.7 × 10^9^; 4.8 × 10^10^)	0.012 *
	Hyperemia	2.1 × 10^10^ (2.1 × 10^10^; 3.0 × 10^9^)	2.9 × 10^10^ (2.8 × 10^9^; 9.5 × 10^10^)	0.638
	ΔII-I (%)	−98% (−100; 864)	−39% (−82; 333)	0.034 *
	*p*-value (II vs. I)	0.008 *	0.049 *	-
	*p*-value (III vs. I)	0.209	0.480	-
Endothelial NO-dependent	Baseline	1.2 × 10^10^ (2.9 × 10^9^; 3.0 × 10^10^)	6.9 × 10^9^ (1.5 × 10^9^; 2.8 × 10^10^)	0.060
	Occlusion	1.5 × 10^10^ (5.7 × 10^9^; 2.9 × 10^10)^	4.0 × 10^9^ (9.8 × 10^8^; 1.9 × 10^10^)	0.015 *
	Hyperemia	2.5 × 10^10^ (3.9 × 10^10^; 5.5 × 10^10^)	1.2 × 10^10^ (8.7 × 10^8^; 4.1 × 10^10^)	0.023 *
	ΔII-I (%)	9% (−72; 1124)	−46% (−84; 90)	0.182
	*p*-value (II vs. I)	0.480	0.071	-
	*p*-value (III vs. I)	0.019 *	0.308	-
Endothelial NO-independent	Baseline	1.1 × 10^10^ (3.1 × 10^9^; 2.5 × 10^10^)	2.4 × 10^9^ (3.3 × 10^8^; 7.7 × 10^9^)	0.002 *
	Occlusion	4.7 × 10^10^ (1.8 × 10^10^; 7.9 × 10^10^)	2.0 × 10^9^ (3.6 × 10^8^; 4.2 × 10^9^)	0.002 *
	Hyperemia	4.6 × 10^10^ (5.2 × 10^9^; 8.9 × 10^10^)	2.2 × 10^9^ (2.2 × 10^8^; 1.1 × 10^10^)	0.002 *
	ΔII-I (%)	293% (36; 2266)	−13% (−75; 59)	0.002 *
	*p*-value (II vs. I)	0.002 *	0.084	-
	*p*-value (III vs. I)	0.010 *	0.754	-

**Table 3:** Relative contribution (%) of the components obtained from the raw PPG signals (* *p* < 0.05).

Component		Test (T)	Contralateral (C)	*p*-Value (T vs. C)
Cardiac	Baseline	58.6 (3.4; 87.8)	63.0 (5.6; 85.9)	0.195
	Occlusion	0.0 (0.0; 1.5)	58.9 (6.6; 91.0)	0.002 *
	Hyperemia	58.7 (14.8; 84.1)	51.8 (5.8; 80.8)	0.182
	ΔII-I (%)	−100 (−100; −76)	−16 (−42; 186)	0.002 *
	*p*-value (II vs. I)	0.002 *	0.530	-
	*p*-value (III vs. I)	0.724	0.308	-
Respiratory	Baseline	3.9 (1.3; 12.8)	5.5 (2.0; 13.0)	0.125
	Occlusion	0.0 (0.0; 2.4)	7.8 (2.1; 20.4)	0.002 *
	Hyperemia	4.1 (2.1; 10.6)	6.5 (2.6; 20.8)	0.031 *
	ΔII-I (%)	−99 (−100; −64)	21 (−61; 282)	0.002 *
	*p*-value (II vs. I)	0.002 *	0.065	-
	*p*-value (III vs. I)	0.875	0.060	-
Myogenic	Baseline	13.2 (3.4; 33.3)	11.2 (2.7; 36.8)	0.894
	Occlusion	0.0 (0.0; 2.1)	15.0 (4.1; 37.1)	0.002 *
	Hyperemia	9.9 (3.4; 30.1)	14.1 (6.2; 43.3)	0.008 *
	ΔII-I (%)	−100 (−100; −84)	33 (−54; 147)	0.002 *
	*p*-value (II vs. I)	0.002 *	0.182	-
	*p*-value (III vs. I)	0.255	0.308	-
Neurogenic	Baseline	10.7 (0.8; 36.6)	7.4 (1.4; 36.4)	0.722
	Occlusion	0.8 (0.5; 9.2)	8.4 (1.0; 33.7)	0.010 *
	Hyperemia	6 (1; 32)	13.7 (4.5; 35.1)	0.019 *
	ΔII-I (%)	−88.2 (−98.3; 180.4)	7 (−76; 101)	0.049 *
	*p*-value (II vs. I)	0.014 *	0.937	-
	*p*-value (III vs. I)	0.195	0.239	-
Endothelial NO-dependent	Baseline	5.5 (1.0; 27.2)	2.1 (0.4; 39.6)	0.158
	Occlusion	21.4 (15.5; 25.9)	1.7 (0.5; 21.3)	0.004 *
	Hyperemia	6.2 (1.1; 16.7)	4.2 (1.9; 26.0)	0.937
	ΔII-I (%)	343 (−27; 1810)	−14 (−84; 225)	0.006 *
	*p*-value (II vs. I)	0.010 *	0.388	-
	*p*-value (III vs. I)	0.754	0.209	-
Endothelial NO-independent	Baseline	4.3 (1.8; 24.6)	1.1 (0.2; 13.7)	0.002 *
	Occlusion	77.6 (59.8; 82.0)	1.1 (0.2; 10.6)	0.002 *
	Hyperemia	12.6 (1.3; 44.7)	0.8 (0.2; 12.3)	0.002 *
	ΔII-I (%)	1759 (286; 4397)	2 (−75; 340)	0.002 *
	*p*-value (II vs. I)	0.002 *	0.753	-
	*p*-value (III vs. I)	0.012 *	0.814	-

**Table 4:** Spearman Rho (*p*-value) correlation between percent contribution and spectral power for each wavelet component (* *p* < 0.05).

Correlation	Test Limb	Contralateral Limb
Δ cardiac % vs. Δ cardiac power	0.839 (0.001 *)	0.930 (<0.001 *)
Δ respiratory % vs. Δ respiratory power	0.943 (<0.001 *)	0.413 (0.183)
Δ myogenic % vs. Δ myogenic power	0.828 (0.001 *)	0.720 (0.008 *)
Δ neurogenic % vs. Δ neurogenic power	0.816 (0.001 *)	0.490 (0.106)
Δ endothelial NOd % vs. Δ endothelial NOd power	0.217 (0.499)	0.637 (0.026 *)
Δ endothelial NOi % vs. Δ endothelial NOi power	−0.357 (0.255)	0.678 (0.015 *)

Absolute power and percent contribution were strongly correlated for the cardiac, respiratory, myogenic, and neurogenic components in the test limb (Rho = 0.83–0.94; all *p* ≤ 0.001), indicating that both metrics captured the same suppression of high-frequency activity during occlusion. In contrast, correlations were weak or absent for the endothelial components (NOd: Rho = 0.217; NOi: Rho = −0.357), reflecting a decoupling between absolute spectral energy and its proportional contribution at very low frequencies. The contralateral limb showed a similar but attenuated pattern, with significant correlations in the cardiac and myogenic bands and moderate correlations in the endothelial components. Together, these results show that absolute and relative metrics converge at high frequencies but diverge for slow endothelial oscillations.

## Data Availability

The data presented in this study are available upon request from the corresponding author.

## References

[1] Park J., Seok H.S., Kim S.S., Shin H. (2022). Photoplethysmogram analysis and applications: An integrative review. Front. Physiol..

[2] Silva H., Rezendes C., Pinto P.C. (2025). Enhancing the quantification of post-occlusive reactive hyperemia: A multimodal optical approach. Pflügers Arch..

[3] Allen J. (2007). Photoplethysmography and its application in clinical physiological measurement. Physiol. Meas..

[4] Zieff G., Stone K., Paterson C., Fryer S., Diana J., Blackwell J., Meyer M.L., Stoner L. (2023). Pulse-wave velocity assessments derived from a simple photoplethysmography device: Agreement with a referent device. Front. Cardiovasc. Med..

[5] Charlton P.H., Allen J., Bailón R., Baker S., Behar J.A., Chen F., Clifford G.D., Clifton D.A., Davies H.J., Ding C. (2023). The 2023 wearable photoplethysmography roadmap. Physiol. Meas..

[6] Silva H., Ferreira H.A., da Silva H.P., Monteiro Rodrigues L. (2018). The venoarteriolar reflex significantly reduces contralateral perfusion as part of the lower limb circulatory homeostasis in vivo. Front. Physiol..

[7] Gil E., Orini M., Bailón R., Vergara J.M., Mainardi L., Laguna P. (2010). Photoplethysmography pulse rate variability as a surrogate measurement of heart rate variability during non-stationary conditions. Physiol. Meas..

[8] Hultman M., Richter F., Larsson M., Strömberg T., Iredahl F., Fredriksson I. (2024). Robust analysis of microcirculatory flowmotion during post-occlusive reactive hyperemia. Microvasc. Res..

[9] Kralj L., Hultman M., Lenasi H. (2024). Wavelet analysis and the cone of influence: Does the cone of influence impact wavelet analysis results?. Appl. Sci..

[10] Silva H., Šorli J., Lenasi H. (2021). Oral glucose load and human cutaneous microcirculation: An insight into flowmotion assessed by wavelet transform. Biology.

[11] Kralj L., Battelino T., Lenasi H. (2025). Effects of oral glucose tolerance test on microvascular and autonomic nervous system regulation in young healthy individuals. Sci. Rep..

[12] Kralj L., Potocnik N., Lenasi H. (2024). Evaluating transient phenomena by wavelet analysis: Early recovery to exercise. Am. J. Physiol. Heart Circ. Physiol..

[13] Stefanovska A., Bračič M. (1999). Physics of the human cardiovascular system. Contemp. Phys..

[14] Kvernmo H.D., Stefanovska A., Kirkebøen K.A., Kvernebo K. (1999). Oscillations in the human cutaneous blood perfusion signal modified by endothelium-dependent and endothelium-independent vasodilators. Microvasc. Res..

[15] Williams B., Mancia G., Spiering W., Rosei E.A., Azizi M., Burnier M., Clement D.L., Coca A., De Simone G., Dominiczak A. (2018). 2018 ESC/ESH guidelines for the management of arterial hypertension. Eur. Heart J..

[16] World Medical Association (2013). Declaration of Helsinki: Ethical principles for medical research involving human subjects. JAMA.

[17] Chuang L.Z.H., Wu L.C., Wang J.H. (2013). Continuous wavelet transform analysis of acceleration signals measured from a wave buoy. Sensors.

[18] Kvandal P., Landsverk S.A., Bernjak A., Stefanovska A., Kvernmo H.D., Kirkebøen K.A. (2006). Low-frequency oscillations of the laser Doppler perfusion signal in human skin. Microvasc. Res..

[19] Silva H., Rocha C., Monteiro Rodrigues L. (2016). Combining laser-doppler flowmetry and photoplethysmography to explore in vivo vascular physiology. J. Biomed. Biopharm. Res..

[20] Rodrigues L.M., Rocha C., Ferreira H., Silva H. (2019). Different lasers reveal different skin microcirculatory flowmotion: Data from the wavelet transform analysis of human hindlimb perfusion. Sci. Rep..

[21] Silva H., Bento M., Vieira H., Monteiro Rodrigues L. (2017). Comparing the spectral components of laser Doppler flowmetry and photoplethysmography signals for the assessment of the vascular response to hyperoxia. J. Biomed. Biopharm. Res..

[22] Rodrigues L.M., Rocha C., Ferreira H.T., Silva H.N. (2020). Lower limb massage in humans increases local perfusion and impacts systemic hemodynamics. J. Appl. Physiol..

[23] Mizeva I., Di Maria C., Frick P., Podtaev S., Allen J. (2015). Quantifying the correlation between photoplethysmography and laser Doppler flowmetry microvascular low-frequency oscillations. J. Biomed. Opt..

[24] Stefanovska A., Poǩorný J. (1997). Correlation integral and frequency analysis of cardiovascular functions. Physiol. Meas..

[25] Mück-Weymann M.E., Albrecht H.P., Hager D., Hiller D., Hornstein O.P., Bauer R.D. (1996). Respiratory-dependent laser-Doppler flux motion in different skin areas and its meaning to autonomic nervous control of the vessels of the skin. Microvasc. Res..

[26] Meyer J.U., Borgström P., Lindbom L., Intaglietta M. (1988). Vasomotion patterns in skeletal muscle arterioles during changes in arterial pressure. Microvasc. Res..

[27] Kastrup J., Bülow J. (1989). Vasomotion in human skin before and after local heating recorded with laser Doppler flowmetry: A method for induction of vasomotion. Int. J. Microcirc..

[28] Söderström T., Stefanovska A., Veber M., Svensson H. (2003). Involvement of sympathetic nerve activity in skin blood flow oscillations in humans. Am. J. Physiol. Heart Circ. Physiol..

[29] Golenhofen K., Bülbring E., Brading A.F., Jones A.W., Tomita T. (1970). Slow rhythms in smooth muscle. Smooth Muscle.

[30] Tankanag A., Krasnikov G., Mizeva I. (2020). A pilot study: Wavelet cross-correlation of cardiovascular oscillations under controlled respiration in humans. Microvasc. Res..

[31] Landsverk S.A., Kvandal P., Kjelstrup T., Benko U., Bernjak A., Stefanovska A., Kvernmo H., Kirkeboen K.A. (2006). Human skin microcirculation after brachial plexus block evaluated by wavelet transform of the laser Doppler flowmetry signal. Anesthesiology.

[32] Tran N., Garcia T., Aniqa M., Ali S., Ally A., Nauli S.M. (2022). Endothelial nitric oxide synthase (eNOS) and the cardiovascular system: In physiology and in disease states. Am. J. Biomed. Sci. Res..

[33] Tóth A., Pal M., Intaglietta M., Johnson P.C. (2007). Contribution of anaerobic metabolism to reactive hyperemia in skeletal muscle. Am. J. Physiol. Heart Circ. Physiol..

[34] Roddie I.C., Shepard J.T., Abboud F.M. (1983). Circulation to skin and adipose tissue. Handbook of Physiology.

[35] Lorenzo S., Minson C.T. (2007). Human cutaneous reactive hyperaemia: Role of BKCa channels and sensory nerves. J. Physiol..

[36] McGarr G.W., Cheung S.S. (2022). Effects of sensory nerve blockade on cutaneous microvascular responses to ischemia–reperfusion injury. Microvasc. Res..

[37] Davis M.J. (2012). Perspective: Physiological role(s) of the vascular myogenic response. Microcirculation.

[38] Kernick D.P., Tooke J.E., Shore A.C. (1999). The biological zero signal in laser Doppler fluximetry: Origins and practical implications. Pflügers Arch.–Eur. J. Physiol..

[39] Silva H., Ferreira H., Rodrigues L.M. (2017). Studying the oscillatory components of human skin microcirculation. Agache’s Measuring the Skin: Non-Invasive Investigations, Physiology, Normal Constants.

[40] Johnson J.M., Kellogg D.L. (2010). Local thermal control of the human cutaneous circulation. J. Appl. Physiol..

[41] Tipton M.J., Harper A., Paton J.F.R., Costello J.T. (2017). The human ventilatory response to stress: Rate or depth?. J. Physiol..

[42] Reynès C., Perez-Martin A., Ennaifer H., Silva H., Knapp Y., Vinet A. (2021). Mechanisms of venoarteriolar reflex in type 2 diabetes with or without peripheral neuropathy. Biology.

[43] Hu H.F., Hsiu H., Sung C.J., Lee C.H. (2017). Combining laser-Doppler flowmetry measurements with spectral analysis to study different microcirculatory effects in human prediabetic and diabetic subjects. Lasers Med. Sci..

[44] Fedorovich A.A., Loktionova Y.I., Zharkikh E.V., Gorshkov A.Y., Korolev A.I., Dadaeva V.A., Drapkina O.M., Zherebtsov E.A. (2022). Skin microcirculation in middle-aged men with newly diagnosed arterial hypertension according to remote laser Doppler flowmetry data. Microvasc. Res..

[45] Jan Y.K., Struck B.D., Foreman R.D., Robinson C. (2009). Wavelet analysis of sacral skin blood flow oscillations to assess soft tissue viability in older adults. Microvasc. Res..

